# Supplementary material for: Allopurinol to reduce cardiovascular morbidity and mortality: A systematic review and meta-analysis
Source: PLoS One. 2021 Dec 2;16(12):e0260844. doi: 10.1371/journal.pone.0260844 (PMC8638940; doi:10.1371/journal.pone.0260844)
Supplement: S1 File — (DOCX) [file pone.0260844.s001.docx]

**S1 File. search string**

**PubMed**

(Hyperuricemia[mesh] OR hyperuricemi*[tiab] OR hyperuricaemi*[tiab]) AND (allopurinol[mesh] OR allopurinol[tiab] OR Uribenz[tiab] OR Allopurin[tiab] OR Allorin[tiab] OR Allpargin[tiab] OR Allural[tiab] OR Pan Quimica[tiab] OR Apulonga[tiab] OR Apurin[tiab] OR Atisuril[tiab] OR Bleminol[tiab] OR Caplenal[tiab] OR Capurate[tiab] OR Cellidrin[tiab] OR Embarin[tiab] OR Suspendol[tiab] OR Foligan[tiab] OR Hamarin[tiab] OR Lopurin[tiab] OR Lysuron[tiab] OR Jenapurinol[tiab] OR Milurit[tiab] OR Milurite[tiab] OR Novopurol[tiab] OR Uripurinol[tiab] OR Urosin[tiab] OR Urtias[tiab] OR Xanthomax[tiab] OR Uridocid[tiab] OR Xanturic[tiab] OR Zygout[tiab] OR Zyloprim[tiab] OR Zyloric[tiab] OR Pureduct[tiab] OR Purinol[tiab] OR Progout[tiab] OR Remid[tiab] OR Rimapurinol[tiab] OR Roucol[tiab] OR Tipuric[tiab] OR Allohexal[tiab] OR Allohexan[tiab] OR Alloprin[tiab] OR "Febuxostat"[Mesh] OR Uloric[tiab]) AND ("Myocardial Infarction"[Mesh] OR Myocardial Infarct*[tiab] OR Stroke*[tiab] OR Heart Attack*[tiab] OR "Stroke"[Mesh] OR Cerebrovascular Accident*[tiab] OR CVA[tiab] OR CVAs[tiab] OR Brain Vascular Accident*[tiab] OR Apoplexy[tiab] OR cardiovascul*[tw] OR ventricul*[tw] OR heart[tw] OR cardiac*[tiab] OR "Quality of Life"[Mesh] OR quality of life[tiab] OR Qol[tiab] OR "Cost-Benefit Analysis"[Mesh] OR Cost Benefit [tiab] OR Cost Effectiveness[tiab] OR Cost-Utility Analys*[tiab] OR Economic Evaluation[tiab] OR Costs and Benefits[tiab] OR Benefits and Costs[tiab] OR "Glomerular Filtration Rate"[Mesh] OR "Glomerular Filtration Rate"[tiab] OR GFR[tiab] OR "Creatinine"[Mesh] OR creatinine[tiab] OR renal function*[tiab] OR kidney function*[tiab] OR "Renal Insufficiency, Chronic"[Mesh] OR Chronic Renal[tiab] OR chronic kidney[tiab] OR "Hospitalization"[Mesh] OR hospitalis*[tiab] OR hospitaliz*[tiab] OR length of stay[tiab] OR "Vasodilation"[Mesh] OR vasodilation[tiab] OR Vascular Endothelium Dependent Relaxation[tiab] OR vasorelaxation[tiab] OR vasodilatation[tiab] OR flow mediated dilation[tiab] OR flow mediated dilatation[tiab] OR "Pulse Wave Analysis"[Mesh] OR Pulse Wave Analys*[tiab] OR Pulse Wave Velocity[tiab] OR Pulse Wave Velocities[tiab] OR Pulse Transit Time[tiab] OR Pulse Transit Times[tiab] OR Pulse Wave Transit Time[tiab] OR (("Tunica Intima"[Mesh] OR "Tunica Media"[Mesh]) AND thickness[tiab]) OR intima media thickness[tiab] OR "Blood Pressure"[Mesh] OR "Blood Pressure Determination"[Mesh] OR mmHg[tiab] OR "Hypercholesterolemia"[Mesh] OR Hypercholesterol*[tiab] OR cholesterol level*[tiab] OR LDL[tiab] OR "Hyperglycemia"[Mesh] OR Hyperglycemia[tiab] OR Hyperglycaemia[tiab] OR glucose level*[tiab] OR "adverse effects" [Subheading] OR adverse effect*[tiab] OR side effect*[tiab] OR adverse event*[tiab])

**Web of science**

| # 3 |  | #2 AND #1  *Indexes=SCI-EXPANDED, SSCI, A&HCI, ESCI Timespan=All years* |
| --- | --- | --- |
| # 1 |  | **TOPIC:** ((Hyperuricemia OR hyperuricemi* OR hyperuricaemi*) AND (allopurinol OR allopurinol OR Uribenz OR Allopurin OR Allorin OR Allpargin OR Allural OR Pan Quimica OR Apulonga OR Apurin OR Atisuril OR Bleminol OR Caplenal OR Capurate OR Cellidrin OR Embarin OR Suspendol OR Foligan OR Hamarin OR Lopurin OR Lysuron OR Jenapurinol OR Milurit OR Milurite OR Novopurol OR Uripurinol OR Urosin OR Urtias OR Xanthomax OR Uridocid OR Xanturic OR Zygout OR Zyloprim OR Zyloric OR Pureduct OR Purinol OR Progout OR Remid OR Rimapurinol OR Roucol OR Tipuric OR Allohexal OR Allohexan OR Alloprin OR "Febuxostat" OR Uloric))  *Indexes=SCI-EXPANDED, SSCI, A&HCI, ESCI Timespan=All years* |
| # 2 |  | **TOPIC:** (("Quality of Life" OR "Cost-Benefit" OR “Cost Effectiveness” OR “Cost-Utility Analys*” OR “Economic Evaluation” OR “Costs and Benefits” OR “Benefits and Costs” OR "Hospitalization" OR hospitalis* OR hospitaliz* OR “length of stay” OR "Vasodilation" OR vasodilation OR “Vascular Endothelium Dependent Relaxation” OR vasorelaxation OR vasodilatation OR “flow mediated dilation” OR “flow mediated dilatation” OR "Pulse Wave Analysis" OR “Pulse Wave Velocity” OR “Pulse Wave Velocities” OR “Pulse Transit Time” OR “Pulse Transit Times” OR “Pulse Wave Transit” Time OR (("Tunica Intima" OR "Tunica Media") AND thickness) OR intima media thickness OR "Blood Pressure" OR mmHg OR "Hypercholesterolemia" OR cholesterol level* OR LDL OR "Hyperglycemia" OR glucose level* OR "adverse effects" [Subheading] OR adverse effect* OR side effect* OR adverse event* OR "Glomerular Filtration Rate" OR "Glomerular Filtration Rate" OR GFR OR creatinine OR “renal function*” OR “kidney function*” OR "Renal Insufficiency" OR “Chronic Renal” OR “chronic kidney” OR “endothelial function” OR “endothelial dysfunction” OR "Myocardial Infarction" OR “Myocardial Infarct*” OR Stroke* OR “Heart Attack*” OR "Stroke" OR Cerebrovascular Accident* OR CVA OR CVAs OR “Brain Vascular Accident*” OR Apoplexy OR cardiovascul* OR ventricul* OR heart OR cardiac*))  *Indexes=SCI-EXPANDED, SSCI, A&HCI, ESCI Timespan=All years* |

**Cochrane library**

search terms: GOUT [tiab] or allopurinol [tiab] or xanthine oxidase inhibitor [tiab]
