# Supplementary material for: Allopurinol to reduce cardiovascular morbidity and mortality: A systematic review and meta-analysis
Source: PLoS One. 2021 Dec 2;16(12):e0260844. doi: 10.1371/journal.pone.0260844 (PMC8638940; doi:10.1371/journal.pone.0260844)
Supplement: S1 Table — a: RCH = retrospective cohort study, CACO = case-control study, RCT = randomized controlled trial, CROSS = crossover study, NA = not applicable, NR = not reported, CM = incidence of cardiovascular mortality, MI = incidence of myocardial infarction, S = incidence of stroke, ALLO = allopurinol, BENZ = benzbromarone, COL = colchicine; b: ALAT = alanine transaminase, AP = angina pectoris, ASAT = aspartate transaminase, BMI = body mass index, BNP = brain natriuretic peptide, BP = blood pressure, BUN = blood urea nitrogen, CKD = chronic kidney disease, Cr = creatinine, CRP = C-reactive protein, eGFR = estimated glomerular filtration rate, HDL = high-density lipoprotein, HF = heart failure, LDL = low-density lipoprotein, LVEF = left ventricular ejection fraction, NSTEMI = non-ST-elevation myocardial infarction, PAD = peripheral artery disease, STEMI = ST-elevation myocardial infarction, UA = uric acid. (DOCX) [file pone.0260844.s002.docx]

| **S1 table a\| Characteristics of the included studies** |
| --- |

| **ArticleID** | **Design** | **# Arms** | **% Male** | **Mean age (yrs)** | **XOI treatment** | **XOI dose (mg/ day)** | **Control** | **Control dose (mg/ day)** | **Duration of treatment (weeks)** | **Follow-up (weeks)** | **% Diabetes** | **% Smoking** | **% Hypertension** | **SBP (mmHg)** | **DBP (mmHg)** | **% Hyperlipidemia** | **Total cholesterol (mmol/ l)** | **% Obesity** | **BMI** | **% Gout** | **Outcomes** |
| --- | --- | --- | --- | --- | --- | --- | --- | --- | --- | --- | --- | --- | --- | --- | --- | --- | --- | --- | --- | --- | --- |
| de Abajo2015 | CACO | 2 | 66/ 67 | 67/ 67 | ALLO | variable | NA | NA | variable | 100 | 19/ 32 | 34/ 43 | 45/ 53 | NR | NR | 31/ 42 | NR | 22/ 25 | NR | 2,8/ 3,5 | CM/ MI/ S/ C |
| Grimaldi-Bensouda2015 | CACO | 2 | 68/ 76 | 60/ 59 | ALLO | variable | NA | NA | variable | NR | 12,4/ 14,5 | 56,8/ 72,7 | 54,5/ 56,3 | NR | NR | 38,1/ 39,9 | NR | 16,9/ 18,9 | NR | 0,9/ 0,6 | CM/ MI/ S/ C |
| Lai2019 | CACO | 2 | 55/ 55 | 65/ 66 | ALLO | variable | NA | NA | variable | NA | 19/ 19 | NR | 78/ 78 | NR | NR | 38/ 38 | NR | NR | NR | NR | CM/ MI/ S/ C |
| Liao2019 | CACO | 2 | 58/ 58 | 77/ 77 | ALLO | variable | NA | NA | NR | NA | 33/ 9 | NR | 86/ 50 | NR | NR | 36/ 17 | NR | NR | NR | NR | CM/ MI/ S/ C |
| Rodríguez-Martín2019 | CACO | 2 | 68/ 71 | 70/ 70 | ALLO | variable | NA | NA | variable | NA | 36/ 25 | 27/ 19 | 73/ 69 | NR | NR | 61/ 52 | NR | 45/ 43 | NR | 17/ 18 | CM/ MI/ S/ C |
| Doehner2002 | CROS | 4 | 100 | 58/ 56/ 68/ 69 | ALLO | 300 | placebo | NA | 0,1/ 1 | 0,1/ 2 | 0/ 0/ NR/ NR | 0/ 0/ NR/ NR | 0/ 0/ NR/ NR | 125/ 118/ 99/ 106 | 78/ 78/ 59/ 65 | 0/ 0/ NR/ NR | NR | NR | 26/ 27/ 27/ 27 | 0 | CM/ MI/ S/ C |
| Nicotero1970 | CROS | 2 | 27 | NR | ALLO | 300 | placebo | NA | 2 | 4 | NR | NR | NR | 134 | 81 | NR | NR | NR | NR | NR | CM/ MI/ S/ C |
| Bayram2015 | PCH | 2 | 47/ 43 | 55/ 60 | ALLO | 300 | usual care | NA | 12 | 12 | 53/ 60 | 23/ 20 | 63/ 63 | 131/ 137 | 76/ 79 | 20/ 13 | 4,9/ 4,8 | mixed | 26,1/ 27,6 | 0 | MI |
| Eliseev2017 | PCH | 1 | 100 | 58 | ALLO | variable | NA | NA | NR | 356 | 18 | NR | 86 | NR | NR | 79 | 6 | 51 | 30.2 | 100 | CM/ MI/ S/ C |
| Kanbay2007 | PCH | 2 | 62/ 71 | 65/ 66 | ALLO | 300 | non-hyper-uricemic individuals | NA | 12 | 12 | 0 | 33/ 48 | 62/ 71 | 133/ 135 | 83/ 80 | NR | 5,0/ 5,1 | mixed | 27,3/ 26,4 | 0 | CM/ MI/ S/ C |
| Tashchuk2017 | PCH | 3 | 83/ 78/ 78 | 51/ 53/ 51 | ALLO | 300 | conventional treatment/ quercetin | NA/ 80 | 26 | 26 | NR | NR | 74/ 83/ 82 | NR | NR | NR | 5,3/ 6,7/ 5,9 | NR | NR | NR | CM/ MI/ S |
| Chen2015a | RCH | 4 | 55/ 57/ 71/ 71 | 43/ 43/ 52/ 52 | ALLO/ BENZ | NR | no treatment | NA | variable | 333 | 0,5/ 0,4/ 1,0/ 1,0 | 67/ 66/ 66/ 66 | 1,7/ 1,3/ 2,6/ 2,6 | 123/ 121/ 135/ 134 | NR | mixed | 5,0/ 5,0/ 5,4/ 5,4 | mixed | 23,4/ 23,2/ 25,6/ 25,6 | 0 | MI |
| Chen2015b | RCH | 4 | 46/ 46/ 63/ 66 | 48/ 48/ 51/ 51 | ALLO/ BENZ | NR | no treatment | NA | variable | 338 | 1,2/ 0,8/ 1,3/ 1,4 | 26,7/ 29,6/ 35,5/ 34, | 2,8/ 1,5/ 3,3/ 3,5 | 124/ 125/ 129/ 130 | 72/ 71/ 76/ 76 | mixed | 5,3/ 5,1/ 5,4/ 5,4 | mixed | 24,2/ 23,4/ 25,1/ 25,1 | 100 | CM |
| Ju2019 | RCH | 2 | 71/ 71 | 71/ 72 | ALLO/ FEB | 100 or 200 or 300/  40 or 80 or 120 | no XOI treatment | NA | NR | 104 (median) | 19/ 18 | NR | NR | NR | NR | NR | NR | NR | NR | 100 | CM/ MI/ S/ C |
| Kim2015 | RCH | 2 | 88/ 88 | 51/ 51 | ALLO/ FEB | variable | untreated hyper-uricemia | NR | NR | 70 | 16/ 20 | 4,2/ 4,4 | 55,1/ 55,9 | NR | NR | 51,1/ 52,2 | NR | 8,4/ 8,7 | NR | 100 | CM/ MI/ S/ C |
| Larsen2016 | RCH | 2 | 72/ 73 | median  64/ 63 | ALLO | variable | no ALLO treatment | NA | NR | 264 | 8,5/ 8,8 | NR | NR | NR | NR | 30,3/ 31,7 | NR | NR | NR | NR | CM/ MI/ S/ C |
| Lin2017 | RCH | 6 | 79/ 79/ 75/ 75/ 83/ 83 | 57/ 57/ 54/ 54/ 54/ 54 | ALLO/ BENZ | variable | no treatment/ benzbromarone | NA/ variable | NR | NR | 29/ 26/ 25/ 25/ 27/ 22 | NR | 61/ 41/ 54/ 37/ 58/ 31 | NR | NR | 44,8/ 38,5/ 51/ 39/ 50/ 37 | NR | NR | NR | 100 | CM/ MI/ S/ C |
| MacIsaac2016 | RCH | 2 | 62/ 62 | 73/ 73 | ALLO | variable | no ALLO treatment | NA | variable | 311 | 24/ 29 | 10/ 10 | 100 | 156/ 157 | 86/ 86 | NR | NR | NR | 26,6/ 28,3 | NR | CM/ MI/ S/ C |
| Ruiz2020 | RCH | 4 | 92 | 61 | ALLO/ FEB | variable | no treatment | NR | NR | 212 | 20 | NR | 51 | NR | NR | 48 | NR | NR | 28.1 | 100 | CM/ MI/ S/ C |
| Singh2016 | RCH | 2 | 45/ 45 | 78/ 79 | ALLO | variable | no ALLO treatment | NA | variable | 101 | 45/ 48 | NR | 86/ 88 | NR | NR | 70/ 73 | NR | NR | NR | NR | S |
| Singh2017b | RCH | 2 | 53/ 54 | age groups reported | ALLO | variable | prior ALLO use | NA | NR | NR | 100 | NR | 86/ 85 | NR | NR | 78/ 81 | NR | NR | NR | 100 | MI/ S |
| Wei2011 | RCH | 2 | 47/ 63 | 72/ 73 | ALLO | variable | no urate lowering therapy | NA | NR | 291 | mixed | NR | mixed | NR | NR | NR | NR | NR | NR | mixed | CM |
| Yen2020 | RCH | 2 | 80/ 80 | 48/ 48 | ALLO/ FEB | NR | no ULT | NR | NR | 234 | 9/ 10 | NR | 31/ 31 | NR | NR | 27/ 27 | NR | NR | NR | 100 | S |
| Pichholiya2016 | RCT | 3 | 67 | 42 | ALLO/ FEB | 300/40 | placebo | NA | 6 | 8 | NR | NR | NR | NR | NR | NR | NR | NR | NR | NR | CM/ MI/ S/ C |
| Badve2020 | RCT | 2 | 62/ 64 | 62/ 63 | ALLO | 100-300 | placebo | NA | 104 | 104 | 57/ 59 | NR | 94/ 96 | 138/ 140 | 77/ 76 | NR | NR | 57/ 59 | 31/ 30 | 0 | MI |
| Borgi2017 | RCT | 3 | 47/ 51/ 51 | 47/ 51/ 51 | ALLO | 300 or 600 | placebo/ probenecid | NA/ 500 or 1000 | 8 | 8 | 0 | 21/ 9/ 10 | 0 | 119/ 119/ 119 | 77/ 78/ 77 | NR | NR | mixed | 33/ 33,4/ 35,7 | NR | S |
| Bowden2013 | RCT | 2 | 75/ 42 | 56/ 62 | ALLO | 300 | placebo | NA | 8 | 8 | 67/ 42 | NR | NR | 142/ 139 | 76/ 74 | NR | 161/ 178 | NR | NR | NR | MI |
| Givertz2015 | RCT | 2 | median  78/ 86 | 63/ 63 | ALLO | 300 or 600 | placebo | NA | 24 | 24 | 52/ 57 | NR | 79/ 77 | 109/ 111 | NR | NR | NR | mixed | 32,6/ 32,0 | 25/ 20 | CM/ MI/ S/ C |
| Goicoechea 2010 | RCT | 2 | NR | 71/ 72 | ALLO | 100 | usual care | NA | 104 | 104 | 36/ 39 | NR | mixed | 146/ 147 | 76/ 77 | NR | NR | NR | NR | NR | CM/ MI/ S/ C |
| Goicoechea 2015 | RCT | 2 | NR | 71/ 72 | ALLO | 100 | usual care | NA | NR | 84 | 36/ 39 | NR | NR | NR | NR | NR | NR | NR | NR | NR | CM/ MI/ S/ C |
| Hosoya2017 | RCT | 4 | 95/ 100/ 98/ 100 | 53/ 50/ 51/ 53/ 51 | ALLO | 200 | topiroxostat/ placebo | 120/ 160/ NA | 16 | 16 | NR | NR | NR | NR | NR | NR | NR | NR | NR | mixed | CM/ MI/ S/ C |
| Huang2017 | RCT | 2 | 60 | 56 | ALLO | 600 | usual care | NA | 4 | 104 | NR | NR | NR | NR | NR | NR | NR | NR | NR | NR |  |
| Jalal2017 | RCT | 2 | 78/ 82 | 59/ 56 | ALLO | 300 | placebo | mixed | 12 | 12 | 61/ 61 | 19/ 16 | NR | 130/ 127 | 78/ 77 | NR | ~5,0 | mixed | 32,9/ 31,7 | 0 | CM/ MI/ S/ C |
| Kanbay2011 | RCT | 3 | 46/ 48/ 53 | 48/ 50/ 54 | ALLO | 300 | normouricemic control subjects/ no treatment | NA/ NA | 16 | 16 | 0 | 0 | 0 | 119/ 123/ 128 | 77/ 76/ 75 | NR | NR | NR | 28,4/ 29,7/ 28,4 | 0 | CM/ MI/ S/ C |
| Liu2015a | RCT | 2 | 46/ 46 | 51/ 50 | ALLO | variable | usual care | NR | 156 | 156 | 100 | 35/ 33 | 0 | 121/ 121 | 74/ 74 | NR | 5,06/ 5,08 | NR | 25/ 25,1 | 0 | CM/ MI/ S/ C |
| Liu2015b | RCT | 2 | 49/ 48 | 51/ 50 | ALLO | variable | usual care | NR | 156 | 156 | 100 | 35/ 36 | 0 | 123/ 121 | 75/ 74 | NR | 5,12/ 5,10 | NR | 25,2/ 25,0 | 0 | CM/ MI/ S/ C |
| Madero2015 | RCT | 2 | 61/ 61 | 47/ 46 | ALLO | 300 | placebo | NA | 4 | 8 | 0 | NR | 0 | 125/ 122 | 84/ 83 | NR | 5,2/ 5,2 | NR | NR | NR | CM/ MI/ S/ C |
| McMullan2017 | RCT | 3 | 47/ 51/ 51 | 41/ 43/ 37 | ALLO | 300 | probenecid/ placebo | 300/ NA | 8 | 8 | 0 | NR | 0 | 119/ 119/ 119 | 78/ 78/ 77 | NR | NR | mixed | 33/ 35,7/ 33,4 | NR | CM/ MI/ S/ C |
| Momeni2010 | RCT | 2 | 45/ 45 | 59/ 56 | ALLO | 100 | placebo | NA | 16 | 16 | 100 | NR | NR | 147/ 145 | 86/ 89 | NR | NR | mixed | 26,8/ 28,9 | NR | CM/ MI/ S/ C |
| Poiley2016 | RCT | 5 | 93/ 96/ 92/ 100/ 96 | 50/ 53/ 53/ 50/ 53 | ALLO (+/ - COL) | 300 | arhalofenate/ placebo | 600/ 800/ NA | 12 | 12 | NR | NR | NR | NR | NR | NR | NR | NR | 33,3/ 31,4/ 32,3/ 32,7/ 31,5 | 100 | CM/ MI/ S/ C |
| Rosenfeld1974 | RCT | 5 | NR | NR | ALLO | NR | placebo | NA | 130 | 130 | NR | NR | variable | variable | variable | NR | NR | NR | NR | 0 | CM/ MI/ S/ C |
| Schumacher 2008 | RCT | 5 | 92/ 93/ 94/ 95/ 94 | 52/ 52/ 51/ 51/ 54 | ALLO | 300 | placebo (/ FEB) | NA/80/120/240 | 28 | 28 | NR | NR | 46/ 46/ 46/ 46/ 52 | NR | NR | 33/ 28/ 34/ 33/ 37 |  | 62 | 32/ 33/ 33/ 33/ 33 | 100 | CM/ MI/ S/ C |
| Segal2015 | RCT | 2 | 32/ 31 | 51/ 51 | ALLO | 300 or 600 | placebo | NA | 4 | 9 | 0 | NR | 100 | 117/ 120 | 74/ 76 | NR | NR | NR | 34,5/ 34,5 | NR | MI/ S |
| Sezer2014 | RCT | 2 | 57 | 66/ 66 | ALLO | variable | no ALLO treatment | NA | 52 | 52 | NR | NR | NR | NR | NR | NR | NR | NR | NR | NR | MI |
| Shi2012 | RCT | 2 | 47/ 62 | 40/ 40 | ALLO | 100-300 | usual care | NA | 26 | 26 | NR | NR | 47/ 43 | 141/ 139 | 87/ 88 | NR | 5,3/ 4,8 | NR | NR | NR | MI/ S |
| Siu2006 | RCT | 2 | 50/ 36 | 49/ 48 | ALLO | 100-300 | usual care | NA | 52 | 52 | 27/ 24 | NR | 73/ 84 | 135/ 138 | 71/ 79 | NR | 5,1/ 6,1 | NR | NR | 0 | C |
| Taheraghdam 2014 | RCT | 2 | 31/ 40 | 67/ 71 | ALLO | 200 | placebo | NA | 12 | 12 | 54/ 71 | 23/ 6 | 31/ 40 | NR | NR | 20/ 17 | 5,0/ 5,1 | NR | NR | NR | CM |
| Takir2015 | RCT | 3 | 10,4/ 33/ 63 | 45/ 50/ 52 | ALLO | 300 | no treatment/ non hyper-uricemic patients | NA | 12 | 12 | 0 | NR | 52/ 61/ 48 | NR | NR | NR | 5,2/ 5,2/ 5,8 | mixed | 30,4/ 30,9/ 30,1 | 0 | CM |
| Taylor2012 | RCT | 2 | 100 | 61/ 57 | ALLO | 300 | placebo | NA | 3/ 4 | 4 | 20/ 15 | NR | 76/ 58 | NR | NR | 56/ 65 | NR | mixed | 32/ 32 | 100 | CM/ MI/ S/ C |
| RCH = retrospective cohort study, CACO = case-control study, RCT=randomized controlled trial, CROSS= crossover study, NA= not applicable, NR = not reported, CM = incidence of cardiovascular mortality, MI = incidence of myocardial infarction, S = incidence of stroke, ALLO= allopurinol, BENZ= benzbromarone, COL= colchicine, | | | | | | | | | | | | | | | | | | | | | |

| **S1 table b\| additional characteristics of trials included in meta-analysis** | | | | | | |
| --- | --- | --- | --- | --- | --- | --- |
| **Article ID** | **Sample size (number of patients)** | **Inclusion Criteria** | **Exclusion Criteria** | **Primary outcomes** | **Secondary outcomes** | **Trial registration** |
| Doehner2002 | 14 | chronic HF; hyperuricemia (>400µmol/L) | history of unstable angina, myocardial infarction, or stroke within 3 months before study; any life-threatening disease including malignancy within the previous 5 years; active myocarditis; serum Cr above 300 µmol/L; severe liver disease (ASAT or ALAT 3 times the upper limit of normal range), gout; a history of allopurinol therapy | postischemic peak leg blood flow after allopurinol therapy | resting arm and leg blood flow, ischemia-stimulated peak arm blood flow, and forearm flow-dependent flow, serum UA, allantoin, adverse events | not reported |
| Nicotero1970 | 18 | hypertension | no hypertensive treatment at baseline | mean BP, pulse, weight, serum UA, urinary UA, serum potassium, serum sodium, Cr clearance, BUN, adverse events | (authors do not differentiate between primary and secondary outcomes) | not reported |
| Pichholiya2016 | 90 | sputum positive tuberculosis patients; age 18-65 years | history of any osteoarthritic condition, taking hyperuricemic drugs, hepatic dysfunction, history of renal calculi, pregnancy lactating females | serum UA | adverse events | not reported |
| Badve2020 | 369 | age ≥ 18 years; CKD stage 3 or 4 (eGFR 15 to 59 mL/min/1.73 m2 inclusive); and random urine albumin to creatinine ratio ≥265 mg/g (≥30 mg/mmol)  Or  evidence of progression of CKD (decrease in eGFR ≥ 3.0 mL/min/1.73 m2 in the preceding ≤ 12 months, calculated as the difference between the first and last tests, based on minimum of 3 blood tests with each test done at least 4 weeks apart). | history of clinically established gout; history of hypersensitivity to allopurinol; kidney transplant recipients; concurrent treatment with azathioprine, 6-mercaptopurine, theophylline,  cyclophosphamide, cyclosporine, probenecid, phenytoin, or chlorpropamide; indication for allopurinol, including tophus or tophi on clinical examination or  imaging study, UA nephropathy, UA nephrolithiasis or urolithiasis; current non-skin cancer malignancy; unresolved acute kidney injury in last 3 months; current pregnancy or breast feeding; any uncontrolled psychological illness or condition which interferes with their ability to understand or comply with the requirements of the study; Elective or imminent initiation of maintenance dialysis or kidney transplantation expected in the next 6 months. | change in the  eGFR from baseline (i.e., randomization) to 104  weeks | composite of a 40% reduction from baseline in eGFR, end-stage kidney disease (dialysis for ≥30 days or kidney transplantation), or death from any cause; a composite of a 30% reduction from baseline in eGFR, end-stage kidney disease, or death from any cause; individual components of the composite kidney outcomes; BP, albuminuria, and serum UA; cardiovascular events; hospitalization for any cause; quality-of-life summary scores on the 36-Item Short-Form Health Survey; adverse events | Australian  New Zealand Clinical Trials Registry number: ACTRN12611000791932 |
| Borgi2017 | 102 | BMI ≥25kg/m^2^; serum UA ≥5 mg/dL | pregnant; history of hypertension; diabetes mellitus, chronic liver disease (or abnormally elevated ASAT/ALAT); coronary heart disease; eGFR <60ml/min/1.73m^2^; kidney stones | flow-mediated, endothelium-dependent vasodilation of the brachial artery | endothelium-independent vasodilation; serum UA; adverse events; homeostatic model assessment of insulin resistance index | not reported |
| Bowden2013 | 24 | CKD; | age <18 years; active illness requiring hospitalization; previous allergic reaction to allopurinol or any of its components; positive blood tests for Hepatitis B and C; dementia; malabsorption syndromes; pregnant; life expectancy <3 months; malignant hypertension; history of medication non-compliance | UA; albumin; inflammatory markers of CRP; Tumor Necrosis Factor-alpha; interleukin-6; triglycerides; total cholesterol; LDL; HDL; total cholesterol/HDL ratio; apolipoprotein B; adverse events | (authors do not differentiate between primary and secondary outcomes) | not reported |
| Givertz2015 | 253 | age 18 years or older; NYHA functional Class II-IV HF due to ischemic or non-ischemic cardiomyopathy; HF symptoms for 3 months despite standard HF treatment with an ACE inhibitor or ARB, and beta-blocker (if tolerated); LVEF ≤ 40% by echocardiography; Serum UA level ≥ 9.5 mg/dl;  At least one of the following additional markers of increased risk:  a. Hospitalization, ER visit or urgent clinic visit for HF requiring IV diuretics within the previous 12 months  b. LVEF ≤ 25%  c. BNP level > 250 pg/ml or NT-proBNP level > 1500 pg/ml | acute coronary syndrome, PCI or CABG within 3 months; VAD or heart transplant likely within the next 6 months; Uncontrolled hypertension (i.e., SBP > 170 mm Hg or DBP > 110 mm Hg); Serum creatinine > 3 mg/dL or estimated GFR < 20 ml/min; Evidence of active hepatitis with ALT and AST greater than 3× normal; Any condition other than HF which could limit the ability to perform a 6-minute walk test (e.g., PAD, orthopedic or neurological conditions); Any diseases other than HF which are likely to alter the patient's global perception of status or quality of life over a period of 6 months; Receiving treatment with allopurinol currently or within 30 days, or having symptomatic hyperuricemia which requires treatment  with allopurinol | composite clinical endpoint that classifies the subject's clinical status as *improved* (Patient Global Assessment moderate or markedly improved), *worsened* (death, hospitalization, ER visit or emergent clinic visit for worsening HF, medication change for worsening HF, Patient Global Assessment moderate or markedly worse), or *unchanged* (neither improved or worsened) at 24 weeks | change in quality of life at and 6-minute walk test at 12 and 24 weeks  (for tertiary outcomes see article) | http://www.clinicaltrials.gov: NCT00987415 |
| Goicoechea2015 | 113 | presence of renal disease (eGFR <60 ml/min); stable clinical condition in terms of no hospitalizations nor cardiovascular events within the 3 months before screening; stable renal function (baseline serum creatinine had not increased by 50% in the 3 months before screening). | history of allopurinol intolerance; already on allopurinol treatment; active infections or inflammatory diseases; HIV infection; chronic hepatopathy; receiving immunosuppressive therapy | hospitalizations; cardiovascular events (defined as myocardial infarction, coronary revascularization or angina pectoris, congestive HF, cerebrovascular disease, and peripheral vascular disease); end-stage renal disease requiring dialysis therapy and/or ≥50% decrease in eGFR; mortality | serum Cr; UA; daily urinary protein excretion; hemoglobin; CRP | not reported |
| Hosoya2017 | 78 | japanese; age 20-64; serum urate level in the run-in period of≥416.4 μmol/L in patients with tophi or a history of gout attacks or ≥535.4 μmol/L in patients with hyperuricemia (however, ≥475.8 μmol/L in patients who were receiving treatment for or had a diagnosis of urolithiasis, hypertension, hyperlipidemia, or diabetes) | onset of gouty arthritis within 2 weeks prior to the start of the study drug administration; primary or secondary hyperuricemia that occurs secondary to specific disorders; HbA1c ≥8.0% or poorly controlled hyperglycemia; renal function impairment (eGFR ≥50 mL/min/1.73 m2); liver impairment (ALT ≥100 U/L and/or AST ≥100 U/L), severe hypertension; use of urate-lowering agents, azathioprine, 6-mercaptoprine, theophylline, the study drug other than topiroxostat, or agents thought to affect the outcomes during the period from 2 weeks prior to the start of the preobservation period until the day of treatment commencement | serum urate reduction | achievement rate of serum urate level ≤356.9 μmol/L; adverse events | not reported |
| Huang2017 | 100 | STEMI, NSTEMI or unstable AP | involvement in other clinical trials, revascularization  surgery within 3 months; hepatic or renal insufficiency;  significant reduction in blood cell counts; allopurinol  allergy | serum creatinine; UA; BNP; blood glucose; blood lipids; malondialdehyde; oxidized LDL; nitric oxide; CRP; tumor necrosis factor alpha; therapeutic effects on angina pectoris and electrocardiogram; cardiovascular events | (authors do not differentiate between primary and secondary outcomes) | not reported |
| Jalal2017 | 80 | age > 18; stage 3 CKD (eGFR 30-59ml/min/1.73m^2^); elevated serum UA levels (7.0 mg/dl for men and 6.0 mg/dl for women) | life expectancy <1 year; uncontrolled hypertension; history of severe liver disease or congestive HF; active infection or on antibiotics; pregnant, breastfeeding, or unwilling to use adequate birth control; history of hospitalization within the last 3 months; expected to undergo living related kidney transplant in the next 6 months; history of immunosuppressive therapy in the last 6 months; history of warfarin use; BMI≥40 kg/m2; serum albumin <3.0 mg/dl. acute gout attacks, receiving or history of adverse reaction to allopurinol. | change in brachial artery flow-mediated dilatation | CRP; IL-6; monocyte chemotactic protein-1; oxidized LDL; serum UA; systolic/diastolic BP; nitroglycerin-mediated dilation; adverse events | C  linicalTrials.gov:  NCT01228903 |
| Kanbay2011 | 72 | age >18 years; asymptomatic hyperuricemia; no presence of diabetes, hypertension, HF, gout, or overt cardiovascular disease | active smokers; patients receiving angiotensin-converting enzyme inhibitors, angiotensin receptor blockers, statins, or supplemental vitamin pills. | endothelial dysfunction; blood pressure; eGFR | fasting blood glucose; serum UA; morning spot urine protein-creatine ratio; hsCRP; LDL; adverse events | not reported |
| Liu2015a | 176 | type 2 diabetes mellitus; urinary albumin excretion rate <20µg/min; age <70 years; good glycaemic control; serum UA 420-476µmol/l after 1 month of low-purine diet; no administration of medications affecting UA metabolism, angiotensin-converting-enzyme inhibitors, angiotensin receptor blockers, or lipid lowering drugs over last 3 months; no disease affecting UA metabolism; | history of gout, primary renal disease, hypertension, severe dislipidemia, acute metabolic disorders in diabetes, malignancy; severe cardiac, hepatic and cerebral disease; | changes in urinary albumin excretion rate, eGFR, serum Cr | adverse events, blood lipids, BP, insulin resistance, incidence of diabetic nephropathy, hypertension | not reported |
| Madero2015 | 72 | age > 18; overweight (body mass index [BMI] >25 kg/m2) or obese (BMI >30 kg/m2); systolic blood pressure >120 to 140 mmHg and diastolic 80 to 90 mmHg; history of high fructose consumption from sources of added sugar (excluding fruits) of >70 g/d | history of diabetes; CKD; liver disease or abnormal liver function tests;  hematologic abnormalities; malignancy; taking any  medications; pregnant | clinic BP | ambulatory BP; weight; triglycerides; total cholesterol; UA; adverse events | clinicalTrials.gov: NCT01157936 |
| McMullan2017 | 102 | BMI ≥25kb/m^2^; serum UA ≥5 mg/dl; no history of hypertension | pregnant; history of diabetes, coronary heart disease, eGFR <60ml/min; active malignancy; history of chronic liver disease or baseline elevation in ALAT/ASAT | kidney specific (renal plasma flow) and systemic RAS-activity (plasma renin activity and ; 24-hour ambulatory BP | serum UA, adverse events | clinicalTrials.gov: NCT01320722 |
| Momeni2010 | 40 | age >18; Type 2 diabetes mellitus; diabetic retinopathy; >500mg/24h urinary protein excretion; bilateral normal sized kidneys; absence of other cause of proteinuria or systemic disease | administration of allopurinol for other indication; serum Cr >3mg/dl; GFR <25ml/min; development of allopurinol side effects | complete blood count; fasting blood glucose; BUN; serum Cr; serum potassium; serum UA; serum ASAT and ALAT; 24-hour urine volume, protein, and creatinine; adverse events | (authors do not differentiate between primary and secondary outcomes) | not reported |
| Poiley2016 | 138 | age 18-75; history of gout with ≥3 flares in 12 months before inclusion; serum UA 7.5-12mg/dl; no urate-lowering therapy or colchicine in 2 preceding weeks | eGFR <60ml/min/1.73m^2^; fractional urate excretion >10%; history of kidney stones; liver function test or creatinine kinase >3x upper limit of normal; secondary hyperuricemia or xanthinuria; uncontrolled hypertension; abnormal electrocardiogram; BMI >42kg/m^2^; medical condition that could interfere with conduct of the study; Concomitant use of potent cytochrome 3A4 inhibitors, cytotoxic drugs, or anticoagulants; long-term treatment with NSAIDs or systemic corticosteroids;  Women of reproductive potential not using contraception | flare incidence | reduction in serum UA; proportion achieving serum UA <6mg/dl; quality of life; adverse events | not reported |
| Rosenfeld1974 | 117 | serum UA >7mg/dl in men or >6mg/dl in women | GFR <40ml/min | serum Cr, Cr clearance | (authors do not differentiate between primary and secondary outcomes) | not reported |
| Schumacher2008 | 402 | age 18-85; history of gout; hyperuricemia (defined for this study as a serum UA ≥8.0 mg/dl); normal (serum Cr ≤1.5 mg/dl) or impaired (serum Cr >1.5 to ≤2.0 mg/dl) renal function at day -2 | intolerance to allopurinol, naproxen, or colchicine; history of renal calculi; alcohol intake of ≥14 drinks/week; hepatic dysfunction with ALAT and ASAT both >1.5 times the upper limit of normal; any other significant medical condition | proportion of subjects with serum UA <6mg/dl at 3 months | proportion of subjects with serum UA <6mg/dl at each visit; percentage reduction in serum UA from baseline; proportion requiring treatment for gout flare; reduction number of tophi; tophi size; adverse events | not reported |
| Segal2015 | 139 | African America; age 18-65; stage 1 hypertension | history of malignant hypertension; total white cell count of less than 2500/mm3, anemia, or thrombocytopenia; history of liver disease; secondary cause of hypertension; known presence of diabetes or fasting blood glucose >126 mg/dL; history of HF, myocardial infarction, cardiovascular disease, or stroke or on a β-blocker or calcium channel blocker for cardiovascular indications, by history, other than for lowering blood pressure; abnormal electrocardiogram requiring acute medical intervention; history of clinical or renal biopsy evidence of renal parenchymal disease; acute gout attack within 2 weeks before enrollment in study; history of drug abuse in the last 2 years, including narcotics, cocaine, or alcohol (>21 drinks/week); an arm circumference >52 cm; history of a reaction to allopurinol or chlorthalidone; pregnancy, lactation, or subjects who are planning to become pregnant during the study period; history of noncompliance, or unable to comply with the study requirements, or who were participating in another study | change in clinic systolic BP | adverse events, serum UA, serum potassium | clinical trials.gov: NCT00241839 |
| Sezer2014 | 96 | age >18 years; eGFR <60ml/min; no hospitalizations or cardiovascular events in last 3 months | history of allopurinol intolerance; ongoing allopurinol treatment; active infections or inflammatory diseases; chronic liver disease; ongoing immunosuppressive therapy | serum UA; potassium; LDL cholesterol; CRP; GFR; renal restrictive index; adverse events | (authors do not differentiate between primary and secondary outcomes) | not reported |
| Shi2012 | 40 | age 18-70; biopsy-prover IgA nephropathy; proteinuria 0.15-2.0 g/24h; serum albumin >3.5g/dl; Scr <3mg/dl; BP <180/100mmHg; serum UA >7mg/dl in men and >6mg/dl in women | received prednisone or immunosuppressive drugs within 2 months prior to randomization; receiving angiotensin-converting enzyme inhibitors and/or angiotensin receptor blocker; allergy to allopurinol; presence of active gout within the past 4 weeks; women who were pregnant or unwilling to use contraception | eGFR | proteinuria; BP; change in blood pressure medication; adverse events | clinicaltrials.gov: NCT00793585 |
| Siu2006 | 54 | renal disease (proteinuria >0.5mg/24h and/or serum Cr >120µmol/l); stable clinical condition in terms of general health and renal function (baseline serum Cr level and daily proteinuria had not increased by >40% within the 3 months before screening) | history of gouty arthritis,  renal stones, and advanced CKD (serum Cr >400 µmol/L); already on allopurinol or azathioprine; history of allopurinol hypersensitivity; women of childbearing age and unwilling to use effective means of contraception; pregnant; lactating women; | *stable renal function* (serum Cr at the end of study that increased by less than 40% compared with baseline); *worsening of renal function* (serum Cr increased by greater than 40% compared with baseline, but not yet requiring dialysis); *end-stage renal disease* (requiring dialysis therapy); *death* | systolic and diastolic BP; urinary protein excretion; hemoglobin level; white blood cell count; platelet count; serum Cr; ALAT; fasting cholesterol; LDL; HDL triglycerides; serum UA; CRP; adverse events | not reported |
| Taheraghdam2014 | 70 | serum UA >6.5 mg/dl for females and >8.2 mg/dl for males; did not receive thrombolytic agents or investigational drugs; admitted within the first 24 h of symptom initiation; | severe poststroke disability; significant comorbidity (i.e. chronic liver or kidney disease, hematologic disease, cancers, frailty likely to cause death within 3 months); probability to make adherence to the study protocol difficult for patients; a previously documented adverse reaction to allopurinol; serum Cr >2.2 mg/dl; gout symptoms; history of recent treatment with allopurinol; strokes secondary to spontaneous brain hemorrhage, trauma, neoplasm, coagulation disorders, aneurysms or arteriovenous malformations; history of regular consumption of iron or antioxidant vitamins during the 4 weeks preceding study involvement. | serum UA; functional status (primary modified  Rankin scale); mortality rates | adverse events | IRCT ID:  IRCT201204299334N1 |
| Takir2015 | 73 | age ≥ 30; serum UA ≥7mg/dL; GFR ≥60 ml/min | presence of diabetes mellitus; history of gout; history of allopurinol use; body mass index ≥35 kg/m^2^; proteinuria ≥ 1gr/ day; presence of autoimmune disease; rheumatology associated disease; presence of hypothyroidism or hyperthyroidism; presence of diseases which may cause chronic inflammation or microalbuminuria (e.g. malignancy, chronic liver disease, hypertension, chronic lung disease) | improvement in insulin resistance defined by homeostatic model assessment of insulin resistance | BP; fasting glucose; fasting insulin; adverse events | clinicalTrials.gov: NCT02008968 |
| ALAT= alanine transaminase; AP= angina pectoris; ASAT= aspartate transaminase; BMI=body mass index; BNP= brain natriuretic peptide; BP= blood pressure; BUN= blood urea nitrogen; CKD= chronic kidney disease; Cr=creatinine; CRP= C-reactive protein; eGFR= estimated glomerular filtration rate; HDL= high-density lipoprotein; HF= heart failure; LDL= low-density lipoprotein; LVEF= left ventricular ejection fraction; NSTEMI=non-ST-elevation myocardial infarction; PAD= peripheral artery disease; STEMI=ST-elevation myocardial infarction; UA= uric acid; | | | | | | |
