# Supplementary material for: Allopurinol to reduce cardiovascular morbidity and mortality: A systematic review and meta-analysis
Source: PLoS One. 2021 Dec 2;16(12):e0260844. doi: 10.1371/journal.pone.0260844 (PMC8638940; doi:10.1371/journal.pone.0260844)
Supplement: S2 Table — a: CACO = case-control study, PCH = prospective cohort study, RCH = retrospective cohort study. (DOCX) [file pone.0260844.s003.docx]

| **S2 Table a\|** Newcastle-Ottawa scale quality assessment for individual cohort and case-control studies | | | | | |
| --- | --- | --- | --- | --- | --- |
| **Article ID** | **Design** | **Selection** | **Comparability** | **Outcome** | **Quality rating** |
| Bayram2015 | PCH | 2 | 0 | 1 | Poor |
| Eliseev2017 | PCH | 1 | 0 | 2 | Poor |
| Kanbay2007 | PCH | 1 | 0 | 1 | Poor |
| Tashchuk2017 | PCH | 1 | 2 | 1 | Poor |
| Chen2015a | RCH | 3 | 2 | 3 | Good |
| Chen2015b | RCH | 3 | 2 | 3 | Good |
| Ju2019 | RCH | 4 | 2 | 2 | Good |
| Kim2015 | RCH | 3 | 2 | 1 | Poor |
| Larsen2016 | RCH | 3 | 2 | 2 | Good |
| Lin2017 | RCH | 4 | 2 | 2 | Good |
| MacIsaac2016 | RCH | 3 | 2 | 3 | Good |
| Ruiz2020 | RCH | 2 | 0 | 3 | Poor |
| Singh2016 | RCH | 3 | 2 | 3 | Good |
| Singh2017 | RCH | 4 | 2 | 3 | Good |
| Wei2011 | RCH | 3 | 2 | 3 | Good |
| Yen2020 | RCH | 3 | 2 | 3 | Good |
| de Abajo2015 | CACO | 4 | 2 | 3 | Good |
| Grimaldi-Bensouda2015 | CACO | 3 | 2 | 3 | Good |
| Lai2019 | CACO | 3 | 2 | 2 | Good |
| Liao2019 | CACO | 3 | 2 | 2 | Good |
| Rodríguez-Martín2019 | CACO | 4 | 2 | 2 | Good |
| CACO = case-control study; PCH = prospective cohort study; RCH = retrospective cohort study | | | | | |

| **S2 Table b\|** Quality assessment for randomized cross-over studies | | | | | | | |
| --- | --- | --- | --- | --- | --- | --- | --- |
| **Article ID** | **Bias arising from randomization process** | **Bias due to deviations from intended interventions (carry-over)** | **Bias due to missing outcome data** | **Bias in measurement of the outcome** | **Bias in selection of the reported results** | **other bias** | **suitability of crossover design** |
| Doehner2002 | Unclear risk | High risk | low risk | unclear risk | unclear risk | low risk | Crossover study for cardiovascular outcomes is less suitable. Furthermore treatment period is very short so no effect of treatment is to be expected during the study |
| Nictero1970 | Unclear risk | unclear risk | unclear risk | unclear risk | unclear risk | low risk |  |
