# Supplementary material for: Allopurinol to reduce cardiovascular morbidity and mortality: A systematic review and meta-analysis
Source: PLoS One. 2021 Dec 2;16(12):e0260844. doi: 10.1371/journal.pone.0260844 (PMC8638940; doi:10.1371/journal.pone.0260844)
Supplement: S3 Table — (DOCX) [file pone.0260844.s004.docx]

**S3 Table. Weight of studies in meta-analysis**

*Cardiovascular mortality*

| **Study name** | **Weight** |
| --- | --- |
| Badve 2020 | 25,94% |
| Borgi 2017 | 1,61% |
| Bowden 2013 | 1,66% |
| Doehner 2002 | 1,65% |
| Givertz 2015 | 19,56% |
| Goicoechea 2015 | 2,70% |
| Hosoya 2017 | 1,62% |
| Huang 2017 | 2,43% |
| Jalal 2017 | 2,44% |
| Kanbay 2011 | 1,62% |
| Liu 2015a | 1,61% |
| Madero 2015 | 1,62% |
| McMullan 2017 | 1,61% |
| Momeni 2010 | 1,64% |
| Nicotero 1970 | 1,64% |
| Pichholiya 2016 | 1,63% |
| Poiley 2016 | 1,62% |
| Rosenfeld 1974 | 1,61% |
| Schumacher 2008 | 1,60% |
| Segal 2015 | 1,61% |
| Sezer 2014 | 1,62% |
| Shi 2012 | 1,64% |
| Siu 2006 | 1,62% |
| Taheraghdam 2014 | 14,44% |
| Takir 2015 | 1,62% |
| Taylor 2012 | 1,63% |

*Myocardial infarction*

| **Study name** | **Weight** |
| --- | --- |
| Badve 2020 | 40,84% |
| Borgi 2017 | 0,77% |
| Bowden 2013 | 0,79% |
| Doehner 2002 | 0,79% |
| Givertz 2015 | 9,33% |
| Goicoechea 2015 | 23,77% |
| Hosoya 2017 | 0,77% |
| Huang 2017 | 2,52% |
| Jalal 2017 | 1,17% |
| Kanbay 2011 | 0,77% |
| Liu 2015a | 0,77% |
| Madero 2015 | 0,77% |
| McMullan 2017 | 0,77% |
| Momeni 2010 | 0,78% |
| Nicotero 1970 | 0,78% |
| Pichholiya 2016 | 0,78% |
| Poiley 2016 | 0,77% |
| Rosenfeld 1974 | 0,77% |
| Schumacher 2008 | 0,77% |
| Segal 2015 | 0,77% |
| Sezer 2014 | 0,77% |
| Shi 2012 | 0,78% |
| Siu 2006 | 0,77% |
| Taheraghdam 2014 | 6,89% |
| Takir 2015 | 0,77% |
| Taylor 2012 | 40,84% |

*Stroke*

| **Study name** | **Weight** |
| --- | --- |
| Badve 2020 | 20,51% |
| Borgi 2017 | 2,27% |
| Bowden 2013 | 2,34% |
| Doehner 2002 | 2,33% |
| Givertz 2015 | 2,26% |
| Goicoechea 2015 | 24,69% |
| Hosoya 2017 | 2,28% |
| Huang 2017 | 2,27% |
| Jalal 2017 | 2,28% |
| Kanbay 2011 | 2,28% |
| Liu 2015a | 2,26% |
| Madero 2015 | 2,28% |
| McMullan 2017 | 2,27% |
| Momeni 2010 | 2,31% |
| Nicotero 1970 | 2,31% |
| Pichholiya 2016 | 2,29% |
| Poiley 2016 | 2,28% |
| Rosenfeld 1974 | 2,27% |
| Schumacher 2008 | 2,26% |
| Segal 2015 | 2,27% |
| Sezer 2014 | 2,28% |
| Shi 2012 | 2,31% |
| Siu 2006 | 2,28% |
| Taheraghdam 2014 | 2,28% |
| Takir 2015 | 2,28% |
| Taylor 2012 | 2,29% |

*Combined outcome*

| **Study name** | **Weight** |
| --- | --- |
| Badve 2020 | 40,84% |
| Borgi 2017 | 0,77% |
| Bowden 2013 | 0,79% |
| Doehner 2002 | 0,79% |
| Givertz 2015 | 9,33% |
| Goicoechea 2015 | 23,77% |
| Hosoya 2017 | 0,77% |
| Huang 2017 | 2,52% |
| Jalal 2017 | 1,17% |
| Kanbay 2011 | 0,77% |
| Liu 2015a | 0,77% |
| Madero 2015 | 0,77% |
| McMullan 2017 | 0,77% |
| Momeni 2010 | 0,78% |
| Nicotero 1970 | 0,78% |
| Pichholiya 2016 | 0,78% |
| Poiley 2016 | 0,77% |
| Rosenfeld 1974 | 0,77% |
| Schumacher 2008 | 0,77% |
| Segal 2015 | 0,77% |
| Sezer 2014 | 0,77% |
| Shi 2012 | 0,78% |
| Siu 2006 | 0,77% |
| Taheraghdam 2014 | 6,89% |
| Takir 2015 | 0,77% |
| Taylor 2012 | 0,78% |
