# Supplementary material for: Allopurinol to reduce cardiovascular morbidity and mortality: A systematic review and meta-analysis
Source: PLoS One. 2021 Dec 2;16(12):e0260844. doi: 10.1371/journal.pone.0260844 (PMC8638940; doi:10.1371/journal.pone.0260844)
Supplement: S1 Fig — Green circle represents low risk of bias; yellow circle represents unclear risk of bias; red circle represents high risk of bias. (DOCX) [file pone.0260844.s005.docx]

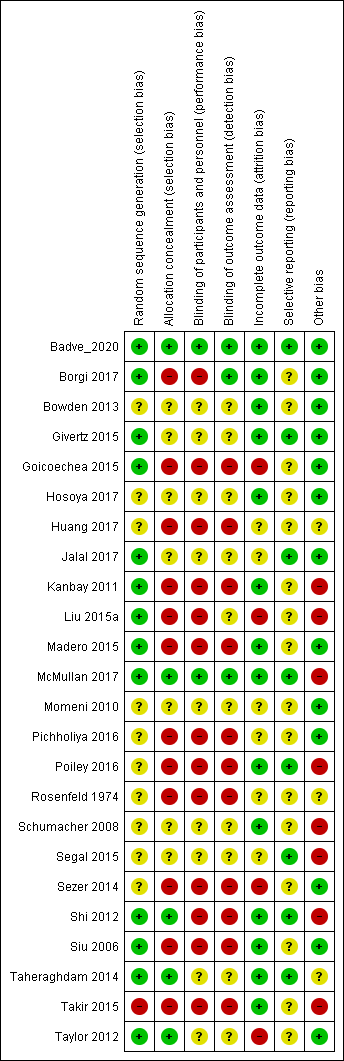


*Green circle represents low risk of bias; yellow circle represents unclear risk of bias; red circle represents high risk of bias;*

**S1 Figure**. Risk of bias of individual RCTs
