# Supplementary material for: Allopurinol to reduce cardiovascular morbidity and mortality: A systematic review and meta-analysis
Source: PLoS One. 2021 Dec 2;16(12):e0260844. doi: 10.1371/journal.pone.0260844 (PMC8638940; doi:10.1371/journal.pone.0260844)
Supplement: S2 Fig — a: Overall effect of allopurinol on cardiovascular mortality: p = 0.738; b: Overall effect of allopurinol on myocardial infarction: p = 0.015; c: Overall effect of allopurinol on stroke: p = 0.993. (DOCX) [file pone.0260844.s006.docx]

**S2 Figure A | Forest plot of cardiovascular mortality**


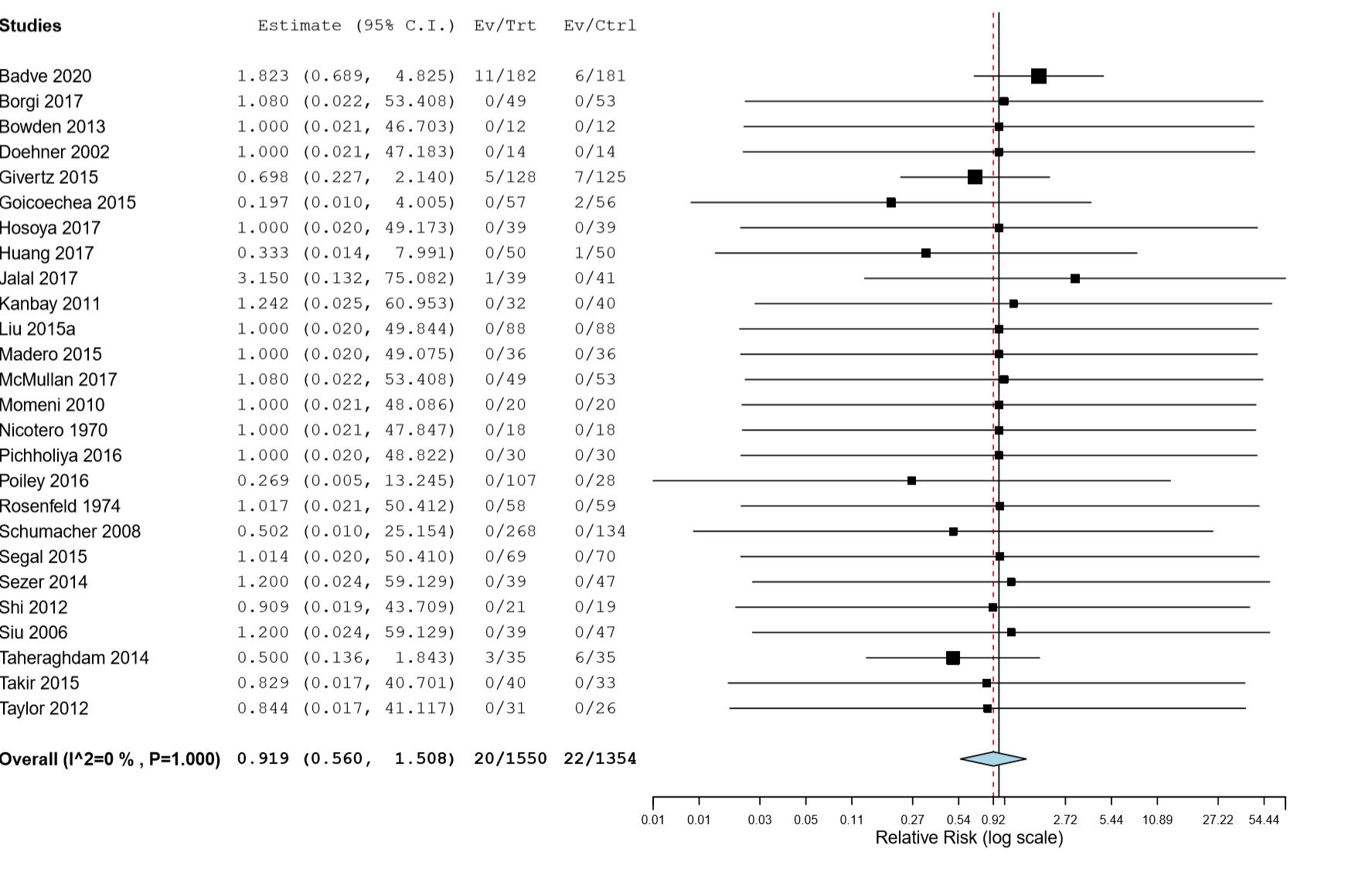


Overall effect of allopurinol on cardiovascular mortality: p=0.738

**S2 Figure B | Forest plot of myocardial infarction**


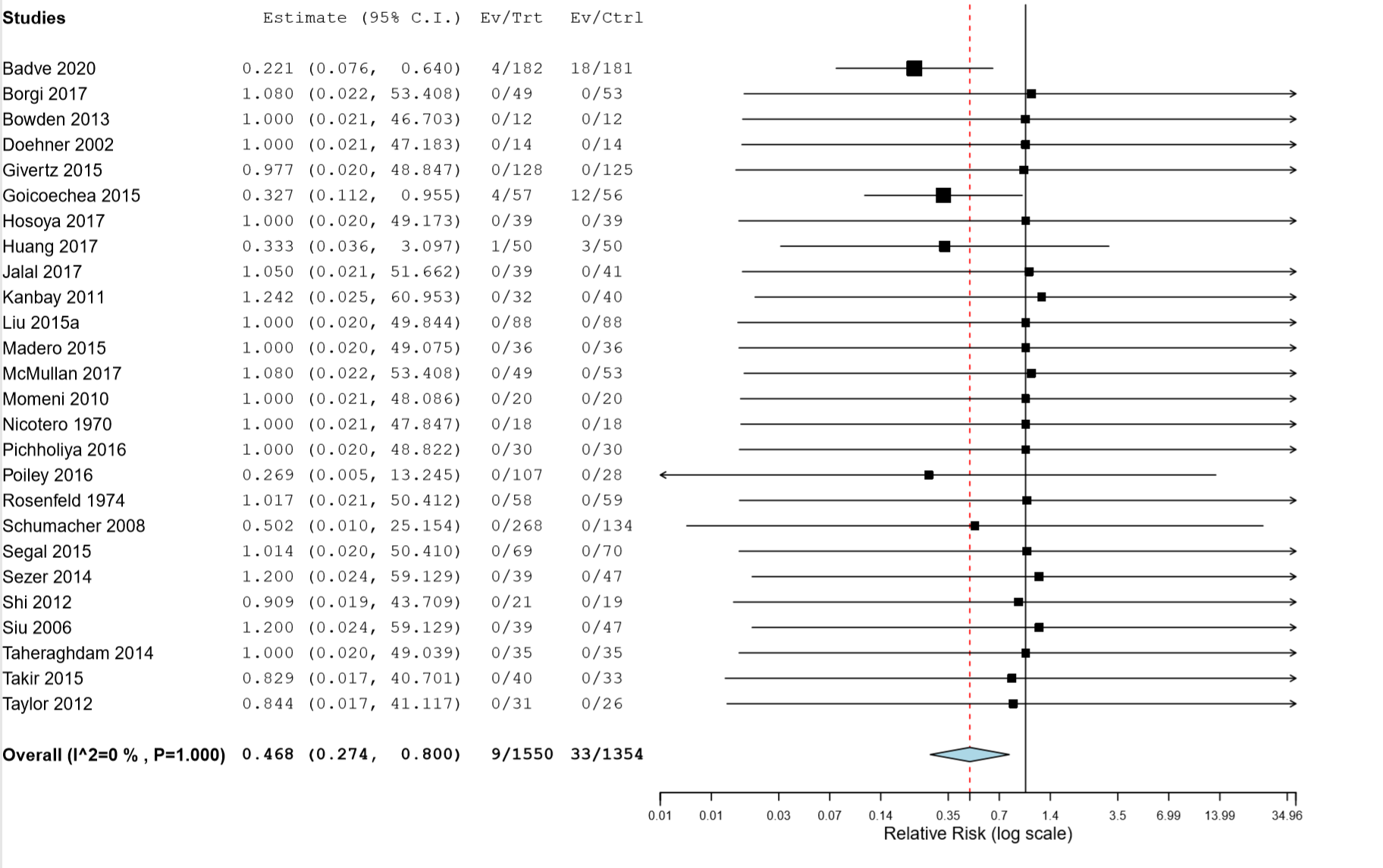


Overall effect of allopurinol on myocardial infarction: p=0.015

**S2 Figure C | Forest plot of stroke.**

*
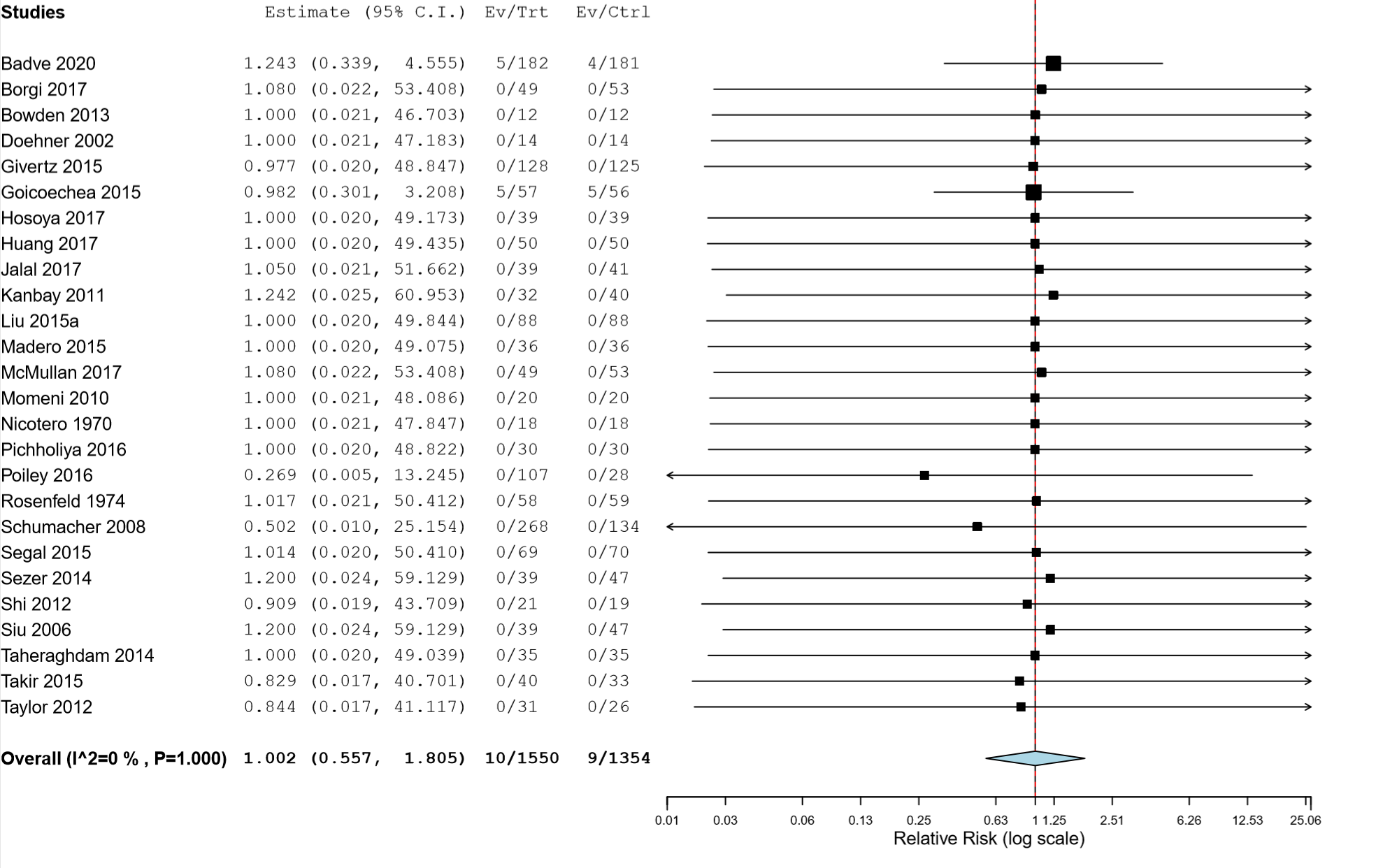
*

Overall effect of allopurinol on stroke: p=0.993
