# Supplementary material for: Allopurinol to reduce cardiovascular morbidity and mortality: A systematic review and meta-analysis
Source: PLoS One. 2021 Dec 2;16(12):e0260844. doi: 10.1371/journal.pone.0260844 (PMC8638940; doi:10.1371/journal.pone.0260844)
Supplement: S3 Fig — a: Egger’s regression P = 0.49; trim and fill +0 studies. Filled circles represent studies included in the meta-analysis, open circles represent studies added during trim and fill analysis; b: Egger’s regression P<0.0001; trim and fill +13 studies. Filled circles represent studies included in the meta-analysis, open circles represent studies added during trim and fill analysis; c: Egger’s regression P = 0.14; trim and fill +12 studies. Filled circles represent studies included in the meta-analysis, open circles represent studies added during trim and fill analysis. (DOCX) [file pone.0260844.s007.docx]

**S3 Figure a. Funnel plot of cardiovascular mortality**


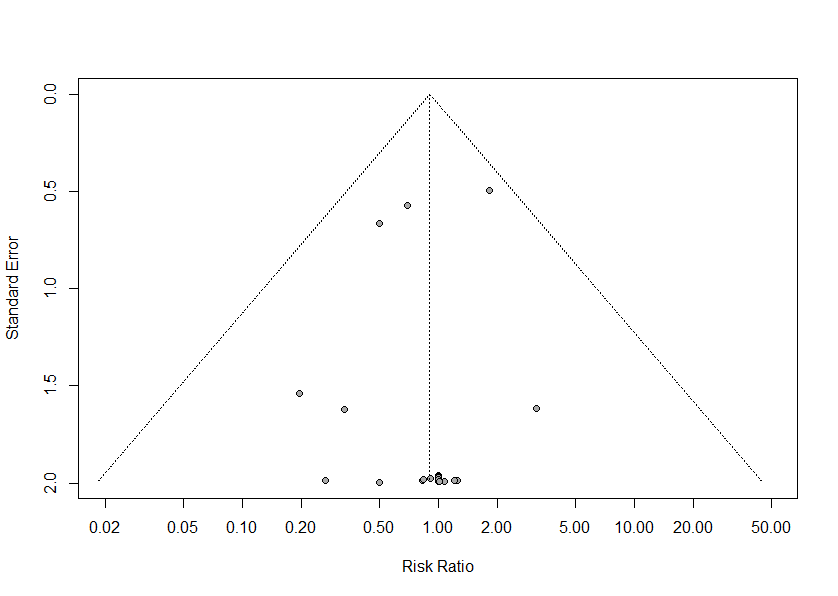


Egger’s regression P=0.49; trim and fill +0 studies. Filled circles represent studies included in the meta-analysis, open circles represent studies added during trim and fill analysis

**
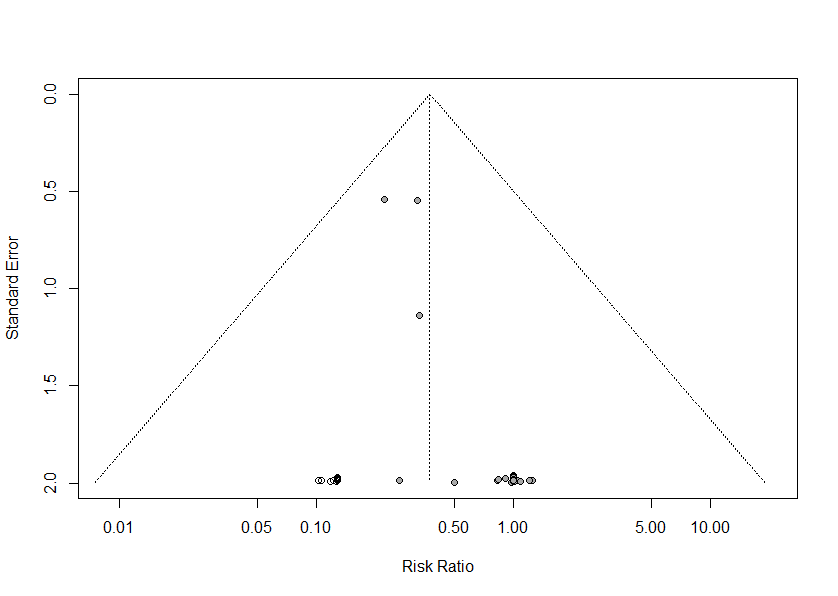
** **S3 Figure b. Funnel plot of myocardial infarction**

Egger’s regression P<0.0001; trim and fill +13 studies. Filled circles represent studies included in the meta-analysis, open circles represent studies added during trim and fill analysis


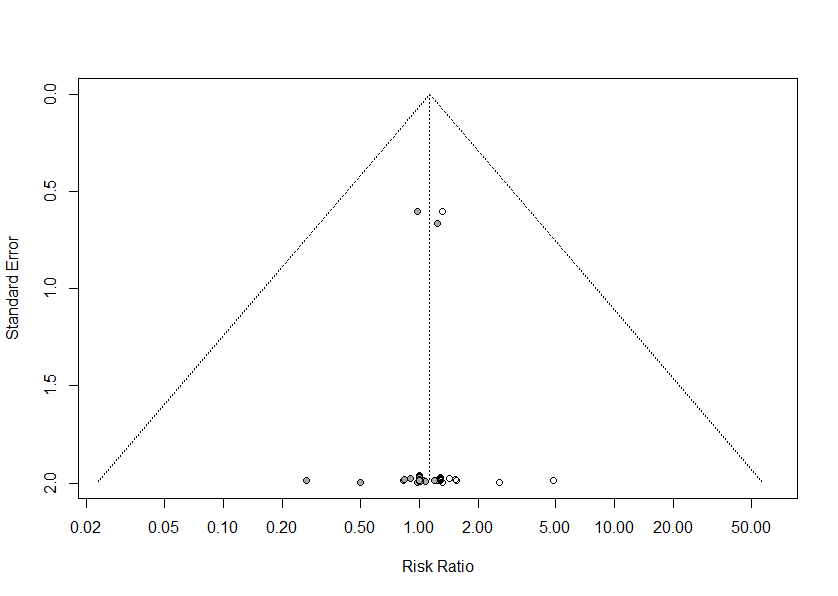
 **S3 Figure c. Funnel plot of stroke**

Egger’s regression P=0.14; trim and fill +12 studies. Filled circles represent studies included in the meta-analysis, open circles represent studies added during trim and fill analysis
